# Secondary Spontaneous Pneumothorax in Asthma: A Dancing Gorilla Moment

**DOI:** 10.7759/cureus.95725

**Published:** 2025-10-30

**Authors:** Maria Kent, Bijaya Prajapati, Christopher Isles

**Affiliations:** 1 General Internal Medicine, Dumfries and Galloway Royal Infirmary, Dumfries, GBR; 2 Respiratory Medicine, Dumfries and Galloway Royal Infirmary, Dumfries, GBR; 3 Acute Internal Medicine, Dumfries and Galloway Royal Infirmary, Dumfries, GBR

**Keywords:** acute medicine, asthma, case report, cognitive bias, imaging, inattentional blindness, respiratory medicine, satisfaction of search, spontaneous pneumothorax

## Abstract

This report describes how cognitive bias contributed to a delay in diagnosing secondary spontaneous pneumothorax in a patient with asthma. A female in her 70s with a past history of asthma presented with shortness of breath at rest and chest pain. She was treated for an acute asthma exacerbation but did not respond as expected. With failure to improve overnight, a review of the chest X-ray revealed a right-sided pneumothorax that had not been previously recognised. This case illustrates how clinicians’ susceptibility to cognitive biases and perceptual errors can affect patient care. It highlights the importance of a systematic approach to interpreting imaging and considering an alternative diagnosis when patients respond poorly to conventional treatment.

## Introduction

Asthma is a prevalent chronic respiratory condition affecting over seven million individuals in the UK [1]. Secondary spontaneous pneumothorax occurs when air enters the pleural space due to an underlying lung disease or pathology. The underlying lung disease causes structural weaknesses, such as bullae or blebs, which may rupture and establish a connection between the alveolar spaces and the pleura. While this complication is well recognised in chronic obstructive pulmonary disease (COPD), it is relatively rare in asthma [2], and asthma was not listed as an underlying lung disease in a consecutive series of 266 patients with secondary spontaneous pneumothorax [3].

The rarity of spontaneous pneumothorax in asthma creates an opening for cognitive bias. Cognitive bias describes shortcuts in mental processing, which can result in errors in judgment. This predominates in type 1 processing - a quick and instinctive mode of thinking as described in the dual process theory [4]. This mode of thinking creates an opening for these shortcuts and results in a higher risk of clinical errors. This report demonstrates how various forms of cognitive bias, including inattentional blindness, confirmation bias, and satisfaction of search, resulted in a delayed diagnosis of pneumothorax in an atypical asthma presentation. Pneumothorax can result in morbidity and mortality, particularly if left unrecognised and appropriate treatment is delayed; therefore, it is essential to recognise these events and address how they can be prevented in the future.

The patient provided written informed consent for the publication of her clinical information.

## Case presentation

A 79-year-old female presented to the Acute Medical Unit with a 10-day history of shortness of breath at rest and wheeze. She described a non-productive cough and had no fever. She had seen her General Practitioner and finished a five-day course of oral antibiotics and steroids, with nebulised salbutamol, but felt no improvement in her symptoms. She also experienced two episodes of sharp central chest pain on the day of admission, with associated nausea and sweating, lasting seconds and worse on deep inspiration. Her past medical history included atrial fibrillation and long-standing asthma that was well controlled with inhaled corticosteroid and long-acting beta-2 agonist inhalers. She described breathlessness on moderate exertion, such as walking uphill, during the previous year, with significant deterioration in her symptoms during the two weeks before admission. She had smoked as a teenager, but not since. 

On examination, she was breathless at rest with a respiratory rate of 21/min and oxygen saturations of 95% on room air. Auscultation revealed bilateral expiratory wheeze but no crackles. 12-lead ECG showed sinus rhythm with a rate of 78 beats per minute and no ischaemic changes. Blood tests, including cardiac troponin and NT pro-BNP, were all normal, except for elevated D-dimer at 1120 ng/ml FEU (normal range <500). Viral swabs were negative. Arterial blood gas on room air showed respiratory alkalosis with hypoxaemia. Peak flow on admission was 150 L/min. The admitting medical team documented the chest X-ray (CXR) as showing linear shadowing in the right lower zone. According to hospital protocol, CXRs are initially interpreted by the requesting team rather than a radiologist, unless a formal radiology report is specifically requested.

**Figure 1:**
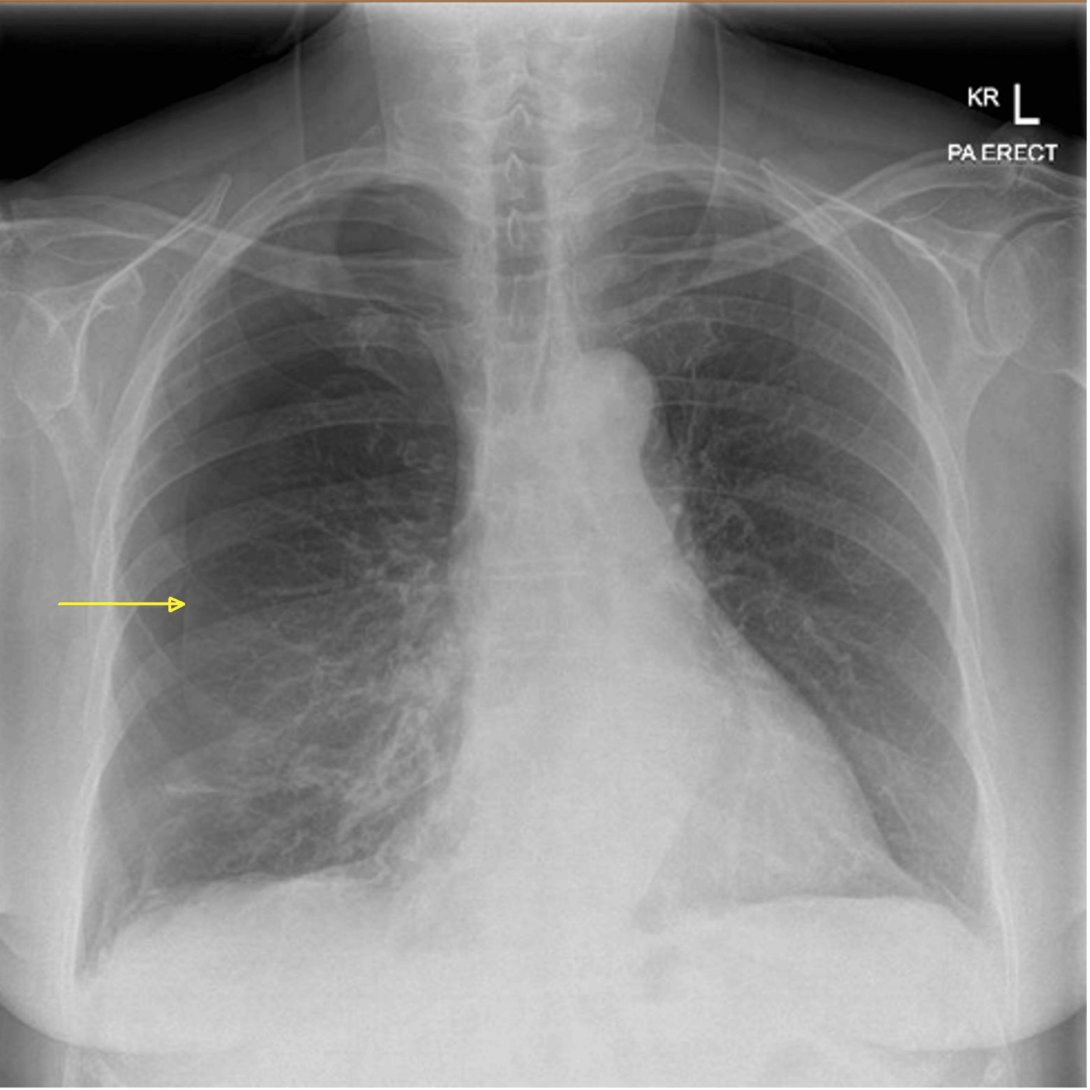
Posteroanterior erect chest X-ray on admission The image shows a right-sided pneumothorax, with the arrow pointing to the lung edge

Based on these findings, the patient was treated as a case of an acute exacerbation of asthma with additional prednisolone, antibiotics, nebulised salbutamol, intravenous magnesium, and oral montelukast. She continued to deteriorate overnight, with worsening breathlessness and increasing oxygen requirement. This prompted a referral to the respiratory team, who reviewed the patient and noted a right-sided pneumothorax on her CXR, which the admitting team had missed (Figure 1).

The CXR showed a large (2.5 cm as measured from the level of the hilum) right-sided pneumothorax, with a visible pleural edge beyond which there were no lung markings. There was no mediastinal shift.

**Figure 2:**
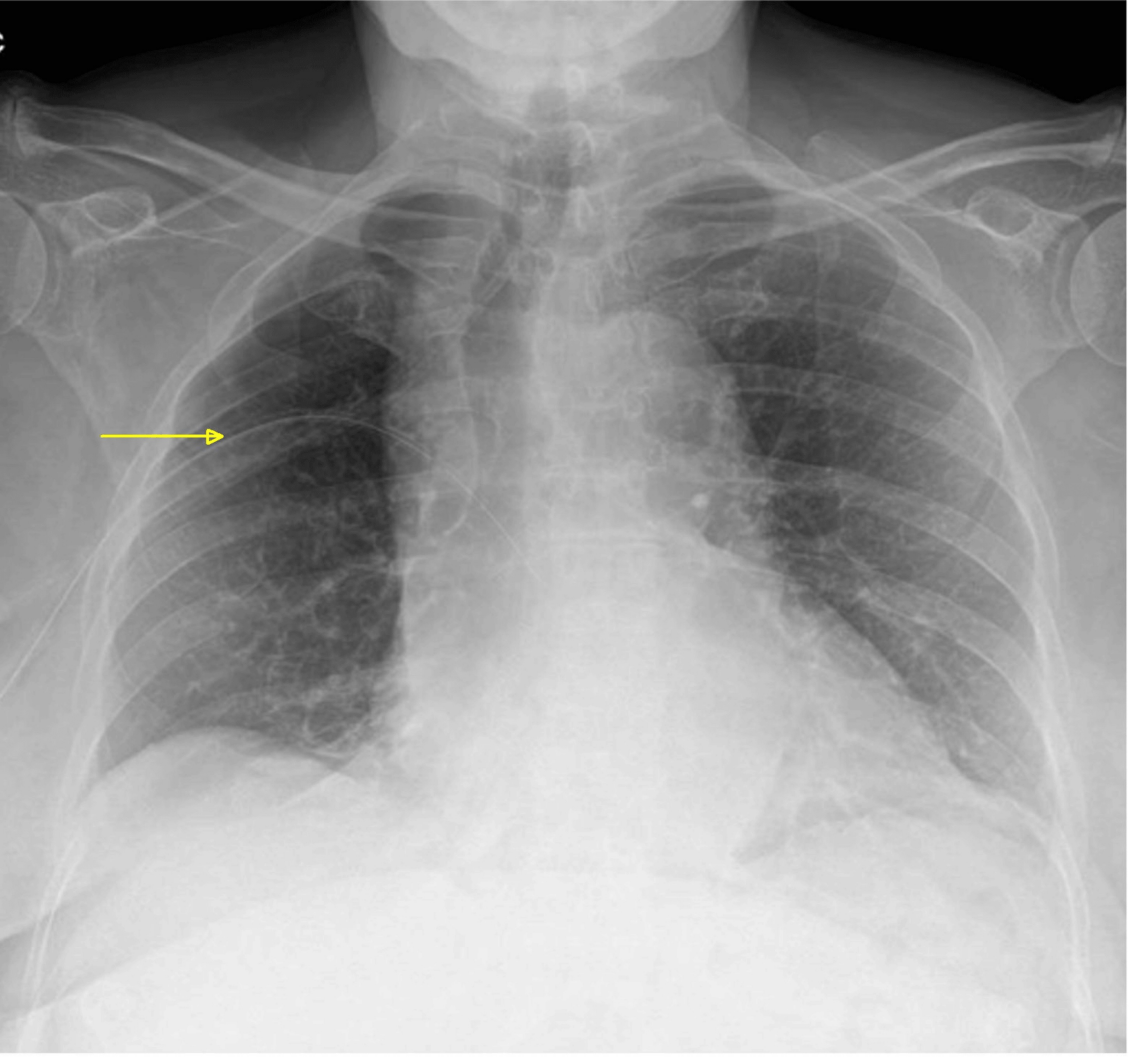
Posteroanterior erect chest X-ray 24 hours following the insertion of chest drain The arrow points to the drain position

The patient was informed of the error and offered an apology, and the incident was formally reported in accordance with the hospital’s guidelines.. The respiratory team recommended a chest drain, which the patient agreed to. The CXR was repeated 24 hours after drain insertion and showed re-expansion of the lung with an appropriate drain position (Figure 2).

The patient’s admission was complicated by hospital-acquired pneumonia, resulting in a total of two weeks in the hospital, but ultimately, she made a full recovery, and when contacted six weeks after discharge, she was pleased to report that her breathing had returned to normal.

## Discussion

Missed diagnostic opportunities (MDOs) can lead to preventable harm for patients. A 2021 longitudinal study of 21 General Practices in England concluded that MDOs occurred in 4.3% of reviewed consultations, with more than a third of these resulting in patient harm. Errors in performing and interpreting diagnostic tests accounted for 35% of these MDOs [5]. Several factors influence a clinician’s interpretation of investigations, including environmental and human factors. This discussion highlights three overlapping phenomena that may have led to the delayed diagnosis in this case: confirmation bias, inattentional blindness, and satisfaction of search.

Since secondary spontaneous pneumothorax is rare in asthma, clinicians may not consider it as a differential diagnosis in asthmatic patients who present with acute shortness of breath, as in this case. Compiling a list of differential diagnoses helps narrow down the most likely cause of a patient’s symptoms; however, it can expose clinicians to confirmation bias, where they may focus on evidence supporting their initial differentials while overlooking information suggesting an alternative cause. In this case, the admitting team used the collected information to generate a list of differential diagnoses, which included acute exacerbation of asthma secondary to infection, as well as pulmonary embolism (PE). Normal ECG, troponin, and NT-proBNP results ruled out a cardiac cause for the patient’s symptoms. The raised D-dimer prompted the suggestion of PE; however, this was considered unlikely given the patient’s adherence to apixaban for atrial fibrillation. Hence, infection-induced acute asthma exacerbation was regarded as the primary diagnosis, supported by the patient’s cough and wheeze, reduced peak flow readings, and arterial blood gas showing respiratory alkalosis with hypoxaemia. Confirmation bias may have come into play, as the presence of normal inflammatory markers, lack of fever, and failure to respond to conventional treatment did not deter the team or prompt consideration of wider differentials.

Simons and Chabris first described inattentional blindness in a 1999 study, where participants were asked to count the number of passes in a basketball game while a woman in a gorilla suit walked through a group of six players. No fewer than 56% of observers failed to notice the ‘gorilla’ while engaged in the primary task of counting passes [6]. These findings led Drew et al. to investigate whether inattentional blindness occurs in expert observers extensively trained in the primary task. In their study, 24 radiologists were given up to three minutes to scroll through each of five lung CT scans in search of lung nodules. Each case contained an average of 10 nodules. The authors inserted a gorilla, 48 times the size of the average nodule, into the left upper zone of the fifth CT. They compared results with a group of 25 naive observers with no medical training who had spent 10 minutes being shown how to identify lung nodules. The results were striking: 83% of the expert radiologists and all the naïve observers missed the gorilla [7]. This represents a classic example of a perceptual error, as described in cognitive psychology, where an individual misinterprets their surrounding environment. This phenomenon came into play in our patient’s case. The clinicians reviewed the chest X-ray with a working diagnosis of infection in mind. They identified a small area of shadowing in the right lower zone, which seemed to support this diagnosis, but consequently overlooked the much larger pneumothorax. 

Another psychological factor contributing to the delayed diagnosis in this patient was the phenomenon of satisfaction of search. In radiological imaging, this occurs when the detection of one lesion leads to another being overlooked. The interpreter comes across a positive finding, in this case the detection of linear shadowing, and therefore stops looking for further lesions [8]. Believing they had identified the cause of the patient’s symptoms, the admitting team ceased further investigation and consequently missed the pneumothorax. This emphasises the importance of having a systematic approach to interpreting images such as chest X-rays, to ensure all aspects have been evaluated and the chance of missed diagnostic opportunities is reduced.

Recognising susceptibility to these biases is the first step in addressing their impact. Cognitive bias awareness training may improve critical thinking and potentially reduce patient harm. There are a variety of practical methods that can be used to overcome cognitive bias [9]. In this case, approaches such as double-reading of images and forming an independent interpretation, without being influenced by another’s opinion, for example, on the chest X-ray, may have been helpful.

## Conclusions

A combination of cognitive bias and perception error contributed to a delay in the diagnosis of secondary pneumothorax in our patient. Confirmation bias led to a focus on results that supported the diagnosis of infection, without considering that this might not account for the full clinical picture. Inattentional blindness occurred as the imaging was reviewed with the working diagnosis of infection in mind, resulting in the large pneumothorax being overlooked. Satisfaction of search resulted in the interpreters looking no further after recognising changes consistent with infection on the X-ray. Cognitive bias can substantially affect patient care; therefore, incorporating cognitive debiasing training into medical education could benefit clinicians and potentially reduce patient harm. Adopting a systematic approach to image interpretation helps ensure that clinically significant abnormalities are not overlooked, and revisiting a diagnosis when there is a poor response to treatment can uncover previously missed information. Ultimately, this report emphasises that greater awareness of cognitive bias and structured reflections on diagnostic reasoning are required to reduce the risk of similar oversights in future practice.

## References

[1] (2025). Asthma and Lung UK: What is asthma?. https://www.asthmaandlung.org.uk/conditions/asthma/what-asthma.

[2] Porpodis K, Zarogoulidis P, Spyratos D (2014). Pneumothorax and asthma. J Thorac Dis.

[3] Onuki T, Ueda S, Yamaoka M (2017). Primary and secondary spontaneous pneumothorax: prevalence, clinical features, and in-hospital mortality. Can Respir J.

[4] Croskerry P, Singhal G, Mamede S (2013). Cognitive debiasing 1: origins of bias and theory of debiasing. BMJ Qual Saf.

[5] Cheraghi-Sohi S, Holland F, Singh H (2021). Incidence, origins and avoidable harm of missed opportunities in diagnosis: longitudinal patient record review in 21 English general practices. BMJ Qual Saf.

[6] Simons DJ, Chabris CF (1999). Gorillas in our midst: sustained inattentional blindness for dynamic events. Perception.

[7] Drew T, Võ ML, Wolfe JM (2013). The invisible gorilla strikes again: sustained inattentional blindness in expert observers. Psychol Sci.

[8] Berbaum KS, Franken EA Jr, Dorfman DD, Miller EM, Caldwell RT, Kuehn DM, Berbaum ML (1998). Role of faulty visual search in the satisfaction of search effect in chest radiography. Acad Radiol.

[9] Croskerry P, Singhal G, Mamede S (2013). Cognitive debiasing 2: impediments to and strategies for change. BMJ Qual Saf.

